# Oxygen, pH, Lactate, and Metabolism—How Old Knowledge and New Insights Might Be Combined for New Wound Treatment

**DOI:** 10.3390/medicina57111190

**Published:** 2021-11-01

**Authors:** Herbert Leopold Haller, Frank Sander, Daniel Popp, Matthias Rapp, Bernd Hartmann, Mehmet Demircan, Sebastian Philipp Nischwitz, Lars Peter Kamolz

**Affiliations:** 1 UKH Linz der AUVA, HLMedConsult, Zehetlandweg 7, 4060 Leonding, Austria; 2 Burn Center, Plastic Surgery of Trauma Hospital Berlin, Warener Strasse 7, 12683 Berlin, Germany; frank.sander@ukb.de (F.S.); bernd.hartmann@ukb.de (B.H.); 3 Department of Surgery, Division of Plastic, Aesthetic and Reconstructive Surgery, Medical University Graz, Auenbruggerplatz 29, 8036 Graz, Austria; daniel.popp@medunigraz.at (D.P.); sebastian.nischwitz@medunigraz.at (S.P.N.); lars.kamolz@medunigraz.at (L.P.K.); 4 Clinic for Orthopedics, Trauma Surgery and Sports Traumatology, Burn Center, Marienhospital Stuttgart, Böheimstraße 37, 70199 Stuttgart, Germany; matthias.rapp@vinzenz.de; 5 Pediatric Intensive Burn Care Unit, Department of Pediatric Surgery, Faculty of Medicine, İnönü University, 44315 Malatya, Turkey; profdemircan@yahoo.com; 6 COREMED—Cooperative Centre for Regenerative Medicine, JOANNEUM RESEARCH Forschungsgsellschaft mbH, 8036 Graz, Austria

**Keywords:** wound, chronic wound, hypoxia, acidosis, alkalosis, lactate, neoangiogenesis, ECM, polylactide

## Abstract

Over time, we have come to recognize a very complex network of physiological changes enabling wound healing. An immunological process enables the body to distinguish damaged cells and begin a cleaning mechanism by separating damaged proteins and cells with matrix metalloproteinases, a complement reaction, and free radicals. A wide variety of cell functions help to rebuild new tissue, dependent on energy provision and oxygen supply. Like in an optimized “bio-reactor,” disturbance can lead to prolonged healing. One of the earliest investigated local factors is the pH of wounds, studied in close relation to the local perfusion, oxygen tension, and lactate concentration. Granulation tissue with the wrong pH can hinder fibroblast and keratinocyte division and proliferation, as well as skin graft takes. Methods for influencing the pH have been tested, such as occlusion and acidification by the topical application of acidic media. In most trials, this has not changed the wound’s pH to an acidic one, but it has reduced the strong alkalinity of deeper or chronic wounds. Energy provision is essential for all repair processes. New insights into the metabolism of cells have changed the definition of lactate from a waste product to an indispensable energy provider in normoxic and hypoxic conditions. Neovascularization depends on oxygen provision and lactate, signaling hypoxic conditions even under normoxic conditions. An appropriate pH is necessary for successful skin grafting; hypoxia can change the pH of wounds. This review describes the close interconnections between the local lactate levels, metabolism, healing mechanisms, and pH. Furthermore, it analyzes and evaluates the different possible ways to support metabolism, such as lactate enhancement and pH adjustment. The aim of wound treatment must be the optimization of all these components. Therefore, the role of lactate and its influence on wound healing in acute and chronic wounds will be assessed.

## 1. Introduction

The prolonged healing of wounds remains an unresolved challenge in modern medicine. Although wounds exhibiting prolonged healing are primarily minor, they have a substantial social impact and influence the patient’s social status, daily living, and professional outcomes. Las Heras et al. [1] estimated 40 million chronic wounds worldwide, and the global wound market is predicted to reach USD 27.8 billion in 2026. The reasons given are the growing prevalence of chronic and surgical wounds as well as burn wounds. Cost-driving components include the increasing use of advanced wound care products, often as first-line therapies, where the advantages of these products over conventional treatment are the driving forces in the market [2]. The advantages may range from greater patient comfort to easier usability. However, the effectiveness of many methods is still questionable, as there may be underlying conditions for the chronification of wounds. Diabetes, frailty, or vascular or immunological diseases even may affect burn wounds [3]. Chronicity in burn wounds is a neglected topic, but Saaiq reported the incidence of Majolins ulcers as between 0.77 and 2%, primarily deriving from the healing per second intention [4].

Furthermore, the mortality of patients with chronic wounds rivals that of cancer patients [1], and the projected outpatient costs range from USD 9.9 to 35.8 billion, as outpatient treatment is a favored modality [5]. In their paper “Publicly Reported Wound Healing Rates: The Fantasy and the Reality,” Fife et al. reported real-world data from randomized controlled trials and from the US Wound Registry that are prone to several risk-stratified quality measures. The conclusion was that RCTs (Randomized Controlled Trials) and US Wound Registry data provided convincing evidence that most wounds did not heal at all, but providers reported online healing rates over healing times that could only be qualified as impossible [6]. Thus, the costs are rising, but the treatment success is stalling.

This article presents the principles of wound physiology and preconditions for successful treatment and healing options to resolve this dilemma with a pragmatic approach. Finally, the possible positive effects of polylactide membranes on pH and wound healing in the neoformation of granulation tissue and graft takes will be demonstrated.

## 2. pH of the Intact Skin

Skin is composed of the well-known epidermis and dermis layers. An “acidic mantle” protects the epidermal layer. Many components influence the secretion of hydrogen ions from the sebaceous glands, and lipids and broad variations in the pH measured have been described [7]. Topical differences might play a role; inconsistent results exist regarding the influence of gender differences, and different ages result in different values along with the influence of hygienic habits and cosmetics. The pH values measured were between 4 and 6 [8].

The acidic mantle pH refers to an intact stratum corneum, where the pH rises towards the basal layers by approximately 2 to 3 pH units [8], and the environment changes to alkaline, with values identical to those for the blood and serum pH in the dermis. During healing and reestablishing the intact stratum corneum, the wound returns to a more neutral or even acidic pH [1]. 

## 3. Wound Healing

### 3.1. Inflammation

In the initial phase of an injury, the damage must be detected, and dead tissue must be resolved and replaced. This starts with an inflammatory phase, where the peripheral nervous system mediates the first reaction to a skin injury, causing “cutaneous neurogenic inflammation” [9,10,11]. From primary sensor neurons, endings from keratinocytes, mast cells, and dendritic cells, the transient receptor channels TRPV1 (transient receptor potential cation channel, subfamily V, member 1) and TRPA1 (transient receptor potential cation channel, subfamily A, member 1) are activated, sending action potentials over the spinal cord, causing pain. In addition, substance P and calcitonin gene-related peptides promote vasodilatation and increased blood flow. 

### 3.2. Hemostasis and Coagulation

Platelets interact with the exposed basement membrane upon damage to the glycocalyx, such as by binding to collagen and activating the coagulation cascade [8,12]. The binding of prothrombin to platelet integrins creates fibrin. Fibrin monomers are formed into protofibrils, which Factor XIIIa stabilizes. Fibrin and the interstitial collagen trap neutrophils and erythrocytes, forming a clot, and collagen I fibers are created under the influence of integrins [13], expressed in fibroblasts, and promote attachment but not multiplication.

### 3.3. Provisional Matrix and the Proliferative Phase

The provisional matrix, composed of fibrin, plasma FN (fibronectin), vitronectin, and platelets, is in contact with migrating keratinocytes of the basal layer on the basal membrane [14]. Initially, migrating epidermal cells find their way between the fibrin clot and the collagen-rich dermis. Thus, the matrix enables cell migration and promotes proto-myofibroblast contraction.

### 3.4. The Proliferative and Regenerative Phase

The proliferative and regenerative phase is characterized by fibroblast migration, collagen synthesis, angiogenesis, granulation tissue formation, re-epithelialization, protrusions developing cell–cell junctions, adhesion by integrins, and traction to the substratum [10,15]. Different cytokines and growth factors such as PDGF, IGF-1, IL- 1β, IL-8, interferon-gamma (IFN-γ), SDF-1, and TNF-α are chemotactic for BMSCs (bone marrow-derived stem cells) and HFSCs (hair-follicle-derived stem cells). MSCs (mesenchymal stem cells) develop immunosuppressive and anti-inflammatory effects, remodel the ECM, and increase angiogenesis and cell differentiation. 

#### 3.4.1. Fibroblasts

Fibroblasts under the influence of PDGF, FGF, EGF, TNFα/β, CTGF (connective tissue growth factor), and IL-1 close wound gaps by producing large amounts of ECM, releasing cytokines such as FGFs, IFNs, TGFβ, VEGF, IGFs, and even hepatocyte growth factor (HGF). The collagen and proteoglycan synthesis are characterized by extracellular matrix formation, structured collagen, bundle regeneration, the degradation of hyaluronic acid, and fibronectin degradation [15]. Promotor-binding sites for corticoids, TGF-β, and retinoids mediate collagen gene expression.

Lactate or a hypoxic environment contributes to gene transcription. It depletes NAD+, which is needed for the production of adenosine diphosphate ribose (ADPR), in which it is converted to NADH.

ADPR is an inhibitor of collagen mRNA’s translation, so the reduction of ADPR increases collagen production. Under the influence of oxygen and iron, ketoglutarate and ascorbic acid protocollagen are hydroxylated to procollagen, forming a helix, and exported from the Golgi apparatus into the extracellular matrix. Further cross-linking and polymerization take place. Together with proteoglycans, they replace the fibrin clot, where the proteoglycan might play a role in assembling collagen fibrils [12,15] with increasing tensile strength.

#### 3.4.2. Keratinocytes

Wound healing also requires closure of the skin defect by keratinocytes. Keratinocytes are mainly stimulated by EGF, KGF, FGF, Il-6, GMCSF (granulocyte-macrophage colony-stimulating factor), MMPs, or activated protein C. They only proliferate in the basal layer, characterized by K5 and K14 intermediate keratin filaments. Cells move through the layers, differentiating by switching the synthesis of K5 and K14 to that of K1 and K10 in the suprabasal layers. Hemidesmosomes and focal adhesions attach the basal layer to the basal membrane, and the suprabasal cells attach by desmosomes, disconnected during the dedifferentiation process to enable the transfer into the upper layers [16]. The lamellar granules in the stratum granulosum contain lipids and proteins, which are transferred to the interstices and formed into high-molecular-weight polymers by cross-linking to envelope proteins, forming a protective barrier.

Later, keratinocytes are dehydrated and flattened, differentiating to terminal corneocytes, interconnected by corneodesmosomes and a lipid layer, which maintains the fluid balance of the epidermis [17]. Epidermal keratinocytes also express protein C and its receptor and activated form, inhibiting apoptosis and upregulating proliferation and migration and MMP-2. In addition, they increase tight junction proteins, inhibit inflammation, and enhance the barrier function [18].

The healing cycle is initiated by the activation of keratinocytes induced by the release of interleukin-1, upregulating the release of K6, K16, and K17 keratins, which might increase the viscoelastic properties and enable cell migration [19], making the keratinocytes contractile, and cause shrinkage of the provisional basement membrane. Activation starts within 24 h upon the change of the keratinocytes to an activated status. Interferon-γ from lymphocytes induces the expression of K17, enabling contractility [17]. According to Safferling et al. [20], keratinocytes move in a shield extension mechanism for a multilayered epithelium, where suprabasal cells never come into contact with the ECM. In the coordination and overlapping of proliferation and migration, epithelium extends and closes the wounds. Basal keratinocytes are deactivated, dermal–epidermal junctions reappear, and hemidesmosomes anchor.

Growth factors, integrins, and the ECM regulate the keratinocyte proliferation in a very complex system during re-epithelialization [19], where EGF, HB-EGF, TGF-α, TGF-β, FGF-10, BMP-6, GM-CSF, IL-1, and TNF-α, and the modulation of microRNAs are involved [21]. MMPs may degrade growth factors and hamper re-epithelialization. Keratinocytes increase growth factors in fibroblasts, which stimulate keratinocyte proliferation retroactively [22]. Fibroblast-derived TGF-β causes the release of K5 and K14, ending the activation of keratinocytes and reverting them to their initial form and function [19]. The regeneration of keratinocytes in human partial-thickness wounds occurs mainly through stem or progenitor cells from the eccrine sweat glands and pilosebaceous units, and through basal stem cells and progenitor cells of the interfollicular dermis, to a lesser extent. On the other hand, regeneration in full-thickness wounds must originate from interfollicular dermal cells from the wound margins.

### 3.5. The Remodeling (Maturation) Phase

During the remodeling phase, circulation is reestablished over the filled gaps, and inflammation stops after a more extended phase of an active metabolic scar. The regeneration of the intact skin depends on local conditions such as delayed wound healing, the wound depth, the tension of the scar, and systemic factors such as hormones or hypertension. Tension in the healing skin results in endothelial dysfunction, promoting ongoing inflammation in the reticular dermis [23], causing pathological scar healing [24] as keloids or hypertrophic scars, persistent pigmentation changes, and fibrosis. Wound contracture is an important part of wound healing [25].

Collagen maturation during the remodeling phase can take 6 to 24 months [26]. The initial collagen matrix comprises 30% collagen type 3, while intact tissue contains 10–20% collagen type 3 and 80–90% collagen 1 [27]. Collagenases and proteases degrade these early fibrils, and this process is paralleled by collagen deposition. Crosslinking mediated by lysyl oxidase increases the thickness and stiffness, and the ratio of collagen 1 and collagen 2 is nearly the same as that for intact connective tissue. Twenty-eight different collagens have been identified in vertebrates; collagens II, III, V, and XI are fibril-forming collagens, while collagen I is the prevalent form in fibrotic conditions. As an essential part of normal wound healing, shrinking is mediated by myofibroblasts, which are generated under the influence of TGF-β. The differentiation into myofibroblasts largely depends on LDH and lactic acid, seems to be pH-dependent, and can be inhibited by gossypol, which inhibits LDH activity [28].

The quality of the regenerated skin is also dependent on the patient’s health conditions, metabolic state, and wound care.

## 4. Aspects of the Pathophysiology of the Skin Injury

Injuries from lacerations of the skin have multiple aspects, including the partial or complete loss of the epidermis, the destruction of the basal membranes as an anchor for the epidermal cells, the corruption of the vascular network and both the superficial and the deep plexus, increased fluid loss as well as temperature and energy loss, and lacerations in deep subdermal tissues [8].

Through the destruction of the local network of vessels, potentially both the superficial and the deep, a hypoxic gradient between the vascularized wound margins and the avascular center of the wound is created [29]. Furthermore, this is linked to increased lactate production, transfer, and local availability, inducing angiogenesis, extracellular matrix generation, fibroblast-induced collagen deposition, and TGF-β synthesis even without acidosis. In addition, the loss of carbon dioxide over the open wound changes the pH locally. Thus, the oxygen tension is diminished because of the vascular lesion, and the presence of necrotic tissue creates a perfect ground for bacteria and infection.

### 4.1. Chronic Wounds

Chronic wounds do not undergo the typical healing path and may stall in one of the healing phases, not closing the skin cover. Not regarding an ongoing discussion over the limitation of the duration of wound healing, wounds with a healing time over more than three months are considered “chronic wounds” [1]. The inability of the host to clear the direct incitement and/or to generate a response to the self-sustaining inflammation program are the two general reasons for chronification.

### 4.2. Persistent Inflammation, an MMP and TIMP Disbalance

The impairment of angiogenesis and dysregulated cytokine and ECM proteins characterize chronic wounds. TGF-β is a controller and modulator of the ECM; it is activated from latent forms, binds to receptors, and releases the ECM components collagen, fibrin, and hyaluronic acid, primarily from fibroblasts [8]. A balanced relationship between free radicals and MMPs enables fighting pathogens and limits tissue destruction. Matrix metalloproteinases together with TIMPs are the main components in wound healing. They cooperate with cells, growth factors, enzymes, and cytokines, and imbalances result in disease [30]. Vasculogenesis, angiogenesis, and other cells form the immature granulation tissue [8].

### 4.3. What Is the Difference between Chronic Wounds and Acute Wounds

Different growth factors and cytokines distinguish each type of wound [31]. The EGF, FGF-2, TGF-β, PDGF, and VEGF levels are increased in acute wounds and decreased in chronic ones. IL-1, IL-6, and TNF-α are increased in acute but also increased in chronic wounds, according to Rayment et al. [32]. The lowest mitogenic activity is attributed to chronic wounds; fibroblasts show signs of premature senescence, MMPs are in excess, and TIMPs are reduced in chronic wounds. With increased ECM degradation and uncontrolled inflammation, the healing time is prolonged.

Keratinocyte behavior in chronic wounds is described as hyperproliferative with impaired migratory potential causing keratosis, or with reduced differentiation or reduced proliferation, and reduced migratory activity [16,33].

### 4.4. Oxygen Tension

Thus, one of the significant features of chronic wound healing is the local oxygen tension, correlating with a pH increase. Oxygen supply is critical for all aspects of wound healing and biochemical energy supply [34,35,36]. Thus, there is an increased energy demand in all regenerative biological processes. In addition, the loss of parts of the vascular network and some necrotic tissue affect the oxygen tension in the center of the wound to the margin, where the center is more hypoxic than the margins. Activated leukocytes in this area show high metabolic demands, so the pH falls as a result of the reduced oxygen tension and lactate accumulates. Hypoxia itself reduces angiogenesis, and angiogenesis rises with increased oxygen tension [35].

Hypoxia as a feature of chronic wounds is generally accepted [2,3,4,5]. The threshold to chronicity is described with a pO_2_ < 20 mm Hg with no healing at all, and a pO_2_ ≥ 40 with complete healing. Between 20 and 40 mm Hg those healed, where a 30° leg elevation had a drop in TcPO_2_ of less than 10 mm Hg [6]. Lactate was demonstrated to be elevated up to 2000% in ischemic skin compared to non-ischemic skin [7].

Lactate accumulation is independent of the oxygen concentration [37,38].

### 4.5. Underlying Mechanism for the Influence of Lactate and pH on Cytokine Production and Enzyme Activities

As both topics are fields of ongoing and extensive research this aspect can only be described incompletely in this paper.

Besides its role as a biomarker and energy transporter, Lactate has a free radical scavenging ability. Lactate affects vasculogenic stem cells via the thioredoxin system [8], fibroblasts, immune cells, and cytokines.

One of the primary mechanisms might be the upregulation of the gene expression as demonstrated in CD4^+^ cells and the upregulation of lactate transporters by lactate accumulation [9]. Baufeld et al. confirmed the ability of Lactate to induce specific genome-wide alterations of gene expression in cultured granulosa cells [10]. Fibroblasts, after Lactate exposure, showed an increase in transcript abundance of genes coding glycolytic enzymes, which showed increased glycolytic enzymes after glucose exposition [11]. The same authors proved a ROS-mediated stabilization of HIF-1α, switching fibroblasts from oxidative phosphorylation to glycolysis metabolism, and an increased transcript load of *MYC*, a human gene increasing the expression of other genes and *SNA/1* (Small Nuclear RNA (snRNA) Associated protein), key facilitators of early somatic cell reprogramming.

pH and pH gradients are essential for proteins like enzymes and connective tissue, and cell metabolism. In addition, pH alterations may change the folding structure, with all mechanical and biological consequences. For example, wound healing is based on the complex cooperation of enzymes and tissue neogenesis, hydroxylation, and other chemical procedures. Therefore a local or a systemic change in pH can profoundly interact with undisturbed wound healing. As in the Lactate metabolism, pH regulates the gene transcription and dysregulated intracellular and extracellular pH dynamics are seen as reason for chronification of wounds and even as a cancer reason [12].

The authors did not find available longitudinal studies on the change of oxygen saturation and lactate levels in wounds during wound healing, but transcutaneous measurement of oxygen saturation is used as a marker for the indication and success of HBOT (Hyper Baric Oxygen Therapy) [13].

### 4.6. Accumulation of Lactate

Lactate accumulation to the 5–10 mM [14] level and, indispensably, oxygen can initiate a healing reaction, potentially via stem cell homing [39]. In addition, lactate accumulation is an indicator of increased metabolic demands, as it is in wounds. Lactate entry and exit in cells is regulated by MCTs 1–4 with a fast flux between cells and circulation, and Lactate accumulation was demonstrated to upregulate Lactate transporters [9]. With lactate in an equilibrium with pyruvate this might regulate the cellular lactate -pyruvate ratio. The availability of LDH and MCTs in most mammalian cells enables them to run the glycolysis and TCA cycle independently [15].

### 4.7. Alkalinity of Wounds

Wound alkalinity, caused by CO_2_, a reduction in oxygen tension, and, possibly, by anion accumulation from bacterial metabolism, is accompanied by lactate accumulation through a Warburg-like effect [40,41]. Lactate at least partially reduces the pH from the alkalotic range to a more physiological range in wounds, enabling cell proliferation and differentiation at a more optimized pH and undisturbed wound healing. Furthermore, the optimal pH range for metalloproteinases for their action is slightly alkaline. In chronic wounds, this compensation mechanism turns out to be insufficient.

### 4.8. Lactate as Fuel for the Cells

Lactate in this situation serves as the “link between glycolytic and aerobic pathway” [42]. Cells with a high energy demand that cannot be fulfilled by oxygen delivery via the circulation turn to lactate metabolism and obtain lactate locally via the lactate pyruvate shuttle. Wounds remain alkaline when the lactate accumulation or the perfusion and oxygen tension cannot reach the physiological ranges.

## 5. Essential Components of Acute and Chronic Wound Healing

### 5.1. Lactate

Lactate was long seen as a waste product of anaerobic metabolism under low oxygen [42]. However, the work of Gertz et al. in 1981 [43] and others revealed that the working heart and muscle simultaneously consume glucose and take up and oxidize lactate in large quantities [44]. Brooks attributed this to at least three crucial functions [45,46].

### 5.2. Lactate as an Energy Source

First, lactate acts as a highly effective essential energy source and recently it was found that lactate uncoupled glycolysis from the TCA cycle and is available as a general energy supply for the cells, with a high flux between cells and the circulating system [15]. It acts as the critical precursor for gluconeogenesis and has a hormone-like function with autocrine, paracrine, and endocrine effects. Because of this, it is referred to as a “lactormon” [42].

### 5.3. Lactate as the Preferred Energy Source

Second, lactate is preferred as a fuel over glucose and fatty acids in brain preparations and humans. Third, intracellular and extracellular lactate shuttles have been described, such as the lactate–peroxisome shuttle or the cytosol mitochondrial shuttle, as enabling lactate exchange between muscles and muscle beds, working skeletal muscles, the brain, the liver, the kidneys, astrocytes, and neurons [47], where lactate might interfere with lipid transfer. This function seems to be disrupted in Alzheimer’s disease [48]. The new understanding of lactate is that of a general energy resource. “The use of lactate as the primary circulating carbohydrate energy source advantageously reserves glucose for particularly vital systems (such as the brain and immune system)” (cited from Rabinowitz et al.) [15].

### 5.4. Lactate Pretending Pseudohypoxia

Third, thus, lactate acts as a signal molecule imitating “pseudo-hypoxia” [11]. Hypoxia is a strong signal to cells, mediating HIF induction and vasculogenesis. Even in a normoxic environment, lactate activates responses, but this usually occurs in a hypoxic environment [38]. It is now seen as the link between glycolytic and aerobic pathways, glycolysis, and mitochondrial respiration and is one of the essential energy-providing and transportation resources. In addition, the Lactate Shuttle Theory establishes its role in cell signaling and the delivery of energy-rich substrates. Lactate can pass cell membranes via the monocarboxylate transporter [49,50,51], and it can enter the mitochondrial reticulum and, in a wide range of cells and components, can additionally lactylate histone–lysine residuals to modify and stimulate gene transcription from chromatin [52], as demonstrated in M1 macrophage polarization.

### 5.5. Lactate Incites Angiogenesis

Lactate also influences cytokines and enzymes with broad metabolic effects. Lactate oxidation into pyruvate depletes the NAD+ pool for ADP-ribosyl transferase, which inhibits the activation of vascular endothelial growth factors. Substrate competition between LDH1 and nuclear poly-ADP ribose polymerase increases procollagen and VEGF transcription in fibroblasts [38]. Studies have demonstrated the effect of lactate on neoangiogenesis and vascularization [53,54,55], even under normoxic conditions.

### 5.6. Lactate Stimulates Collagen Synthesis and the Synthesis of ECM

Lactate stimulates collagen production in fibroblasts [56]. Liu demonstrated that the products of glycolysis increased HIF-1α, a transcriptional factor modulating the genetic reaction to hypoxia [57]. Vural [58] demonstrated that HIF increases the healing of wounds, stimulates VEGF, and promotes angiogenesis. Furthermore, myeloid HIF-knockout mice showed significantly impaired take rates for skin grafts. Trabold postulated that lactate might be a surrogate for hypoxia, initiating the healing mechanism [38].

### 5.7. Lactate Is a Radical Scavenger

Free radicals diminish LDH.

Relatively recently, the role of lactate as a radical scavenger was discovered to be effective in reducing oxidative stress and in the early control of inflammation [59,60]. A close relationship with redox reactions occurs in the endoplasmic reticulum, where Fe^+++,++^ forms chelates with lactate, which enhances OH^−^ in the presence of H_2_O_2_.

### 5.8. Lactate Toxicity

Hunt et al. described the toxicity of lactate at higher levels with a decrease in VEGF production, and Beckert stated that a 15 mM lactate concentration in HUVECs did not reveal visible evidence of toxicity, while VEGF production increased for more than 24 h [61].

## 6. The Role of TGF-β

TGF-β is one of the most potent cytokines in wound healing. However, there is general agreement on the lack of all the TGF-β isoforms [62] in chronic wounds. Nevertheless, controversial observations on different isoform amounts in ulcers of different origins and epithelial cells in different locations have been published. There is agreement on an overall decreased TGF-β levels in chronic wounds, but the TGF-β levels are high in the keratinocytes [62]. Thus, although TGF-β function is critical for undisturbed wound healing, it seems as though the absolute levels of isoforms are not crucial. The temporal coordination and the different cell targets did not allow us, until now, to define the exact role of TGF-β in skin healing; furthermore, different effects on different compartments and cell types must be addressed.

Liarte [62] pointed out some fundamental aspects of the complex TGF-β network. The continuous exposure of keratinocytes to TGF-β influences the evolution and alterations of the cell cycle response in keratinocytes, influencing changes in keratin expression by modulating gene expression and the induction of the mechanism related to epithelial mesenchymal transition.

However, many relevant challenges, including keratinocyte transdifferentiation, a lack of scientific reasoning, a possible sensitization to TGF-β during the healing process, and the autocrine potential of epidermal cells, necessitate ongoing research. In addition, long-term elevated TGF-β might contribute to a closed feedback loop in the context of stagnated re-epithelialization. However, TGF-β levels show controversial results in acute and chronic wounds. They are elevated in acute wounds and reduced in chronic wounds, with elevated levels in the epidermis [62], where the persistence of receptor expression might result in a “feedback loop,” resulting in a fibrotic phenotype of fibroblasts. Furthermore, TGF-β is closely linked to fibrosis [10,63]. Moreover, it is involved in wound healing, angiogenesis, inflammatory processes, collagen synthesis and deposition, and the modeling of the extracellular matrix [33]. Three isoforms are secreted in inactive forms as an LLC (large latent complex) to the ECM. They can be activated by an integrin-dependent pathway, including proteases and metalloproteases, changes in pH, the denaturation of LAP, reactive oxygen species, and thrombospondin-1 [64,65,66], where TGF-β can be activated.

TGF-β directs leukocyte migration in the wound together with PDGF, IL-1, and other cytokine complexes that are translocated into the nucleus, by activating the SMAD pathway, by which the expression of various genes is regulated [67].

In addition, it initiates the transformation of monocytes into macrophages, together with ECM and complement fragments, clearing the debris and initiating the formation of granulation tissue [68]. TGF-α stimulates keratinocyte migration and proliferation, neoepithelial formation and differentiation, and the basement membranes’ neoformation, while macrophage-released TGF-β and VEGF are active in angiogenesis [19].

## 7. Methods to Improve Wound Healing

### 7.1. Effects of Topically Applied Lactate

Several authors have tested the topical application of lactate.

#### 7.1.1. Trabold et al.

Trabold et al. [38] performed experiments on rats with polyglactin 910 (Vicryl). Lactate increased the VEGF level on Day 7, with statistical significance on Days 14, 18, and 21. In addition, TGF-β was elevated twofold in the treated group. IGF-1 (insulin-like growth factor) was equal to the controls on Day 7 but declined rapidly at Days 14, 28, and 21. IL-1β was significantly elevated on Day 7 but did not show a difference from control on all the other days.

#### 7.1.2. Rendl et al.

Rendl et al. [69] tested a topical cream containing lactic acid on epidermal equivalents. They found differentially modulated vascular endothelial growth factor (VEGF), angiogenin (ANG), and interleukin (IL)-8 secretion by human epidermal equivalents. VEGF excretion was increased with creams having concentrations of 1.5 and 3% but not with a cream containing 5%. IL-8 was not significantly changed, and ANG (angiogenin) decreased with increasing concentrations. Increasing concentrations led to increased apoptosis and cell death.

#### 7.1.3. Porporato et al.

Porporato et al. [70] tested lactate delivered from Matrigel, polylactide (poly-L-lactide), and poly-D, L-lactide-co-glycolide lactide in mice in an ischemia–reperfusion model. They demonstrated that lactate–glycolide combinations could be used for a rate-controlled lactate release, and they envisioned a future for lactate treatment for the improvement of wound healing in the development of a formulation with sustained lactate release in adapted amounts. In addition, the angiogenesis inhibitor SU5416 prevented the stimulation of PLGA-stimulated wound healing [71]. 

#### 7.1.4. Hunt et al.

Hunt et al. [35] tested topically applied lactate in Matrigel. In a first experiment, they tested whether lactated Matrigel induced angiogenesis, and in a second, whether there existed a quantitative relationship between lactate monomers and VEGF. In a third experiment, they tested the inhibition of lactate’s effects by oxamate, an inhibitor of LDH and ADP ribosylation. Their first experiment proved angiogenesis in the lactate-containing Matrigel probes, whereas no angiogenesis in lactate-lacking or insoluble lactate preparations occurred. The second experiment demonstrated a direct relationship between VEGF and lactate in a range below that of lactate toxicity. In the toxic range, VEGF fell significantly. In the third experiment, oxamate reduced angiogenesis.

#### 7.1.5. Ring et al.

In mice, Ring et al. [55] used a dorsal skinfold chamber to analyze the angiogenic response to a porous lacto-capromer terpolymer dermis substitute. Quantitative analysis showed a significantly increased vessel density on Days 5 and 10 at the margin of the implant, and intravital microscopy showed a perfused microvessel network at the borders, while the surrounding tissue did not change. In another trial, they compared an ε-caprolactone terpolymer matrix with a pore size of 50–400 µm to a PEGT/PBT (polyethylene glycol terephthalate/polybutylene terephthalate) block copolymer with a pore size of 250–300 µm. Both polymers induced a solid angiogenic response, but the functional vessel density was 1.3 times higher for the ε-caprolactone terpolymer on Day 10 [53,55].

#### 7.1.6. Gürünlüoglu et al.

Gürünlüoglu et al. from the research team of Prof. Demircan, applied a topical lactate application using a polylactide membrane (Suprathel) on partial-thickness burn wounds in children and tested the systemic and local effects. In a wet environment, the polylactides degraded to monolactides, releasing lactic acid and salts in physiological buffers.

They tested the effects of polylactide membranes, Hydrofiber Ag, and a control on the telomerase expression and skin quality in partial-thickness burns [72]. They found an increase in telomerase expression after PLM treatment. In addition, the absolute cell count in the healed epidermis was higher after PLM treatment. Both effects were hypothesized to be attributable to lactate and its radical-scavenging effects.

In a prospective and randomized study, they compared the parameters of systemic oxidative stress to those with a hydrofiber-silver dressing and a control [73]. They tested the TOC (total oxidant capacity), TAC (total antioxidant capacity), MDA (serum malonaldehyde), and GSH (glutathione) levels. PLM use reduced the TOC values to normal levels, the TAC levels were increased, and the drop in TOC was faster in the PLM group than in the comparison group.

In a third prospective and randomized study, [74] evaluated the levels of different biomarkers in children with partial-thickness burns, comparing PLM to hydrofiber Ag and a control. They found an early decrease in IL-6 and TNF-α in both serum and tissue samples in the PLM-treated group during the first days, compared to the HF-Ag group, while TGF-β was increased in this group for three weeks. Thus, they concluded that PLM controlled inflammation earlier, both systemically and in burnt tissue, and the early and time-limited increase in TGF-β might prevent hypertrophic scarring.

### 7.2. Topical Acidification

#### 7.2.1. Leveen et al.

“Acidification” was tested by Leveen et al. [75] with 7% Carbopol, 20% polyacrylic acid, and 1% acetic acid to determine the ammonium-binding capacity and oxygen tension under changing pH conditions in 154 wounds and 27 split-thickness skin grafts, where the pH of healing wounds was compared to that of non-healing wounds, and the presence of liberated ammonia was determined. Their studies revealed that the failure of wound healing was correlated to an alkaline pH in the wound. In addition, wounds exposed to open air lost CO_2_ rapidly and established local metabolic alkalosis. The pH of open wounds was 7.6 or higher. Only 19 out of 137 wounds showed a pH of 7.4 or below. In addition, 27 skin grafts failed at pH levels created by active ammonia production, which was determined in 137 wounds.

The potential for changing the pH was tested in 1% acetic acid, Aserbine, 4% Carbapol (a polymer from acrylic acid), 15% acrylic acid, and 20% acrylic acid. Acrylic acid reduced the pH to around 5, and it reverted to the initial level after approximately 1.5 h. Aserbine resulted in the same initial pH drop, but it was reversed to the initial value after 2 to 3 h, and the polyacrylic solutions caused more significant pH drops and recurrence to the initial pH values after 4 h, from pH 8 to 7 in the 15% solution and from pH 5 to 6 after the same time. No information on the healing rates under the topical treatment with either substance was provided. In another study, Aserbine did not improve the healing time compared to SSD (silver sulfadiazine) or Daromide, a mercurochrome-containing ointment, and the infection rate was the lowest for SSD [76].

#### 7.2.2. Kaufman et al.

They [77] performed a randomized controlled animal study with guinea pigs on the topical application of three buffered solutions of pH 3.5 (citric acid), 7.42 (tris base), and 8.5 (tris base) in partial-thickness burns. They used irrigation disks with 0.15 mL/cm^2^ for irrigation once in 24 h and one control without irrigation of the disk. The dressings were changed every seven days, and the wounds were assessed. The wounds treated with pH 3.5 solution epithelialized the fastest and epithelialized faster at pH 3.5 and 7.42 than at 8.5 and the non-irrigated wounds. On Day 21, 62.5% of the pH-3.5-treated wounds were healed, while only 12.5% were healed in the other groups. The contraction rate did not differ between the groups, nor did the scar thickness, except in the alkalinity group compared to the neutral pH group. This study evaluated the pH of the irrigation fluid immediately after application and not at the end of the 24 h before further irrigation, so the final pH of the wounds remains unclear.

#### 7.2.3. Strohal et al. 

They [78] treated 30 patients with critically infected chronic leg ulcers with an acid-oxidizing solution. They reduced the highly alkalotic pH to a less alkalotic one. In their study, the mean pH of 9.25 ± 0.61 of the chronic wounds on Day 0 was reduced to 7.28 ± 0.71. Thus, a highly significant decrease in wound size was observed at the end of the study period.

#### 7.2.4. Smith et al.

Different organic and inorganic acids such as acetic acid or hypochlorite can act effectively as antimicrobial agents, acidifying wounds during and after application. Smith [79] used hypochlorite solution with a pH of 8.6, which decreased the bacteria/cm^2^ by 1 or 2 log 10. However, acidic tap water with a pH of 5.7 showed mixed effects. NaOCl concentrations higher than 0.01 were cytotoxic to fibroblasts [80].

### 7.3. Reducing the Bioburden

Reducing the bioburden might promote wound healing if the pH does not affect keratinocytes and fibroblasts in a cytotoxic range. In addition, the more acidic pH might support oxygen release in the wound and improve wound healing by activating neovascularization and collagen synthesis in cooperation with lactate and HIF-α. Angiogenesis and collagen deposition tend to be diminished by hypoxia [81]. Lactate signals simulate hypoxia even under normoxic conditions. When initiated under hypoxic conditions, the signal remains until the newly created vascularization fulfils metabolic needs. Kaufman concluded that a strongly acidic pH did not reduce the migratory speed of epidermal cells [77].

#### 7.3.1. Gethin et al.

Gethin et al. [82] used topical manuka honey because of its acidity for the treatment of 20 chronic ulcers. After two weeks, they found a statistically significant decrease in pH. Wounds with a pH > 8 did not decrease in size, while wounds with a pH ≤ 7.6 showed a 30% decrease in size.

#### 7.3.2. Silvetti et al.

Silvetti [83], cited by Kaufman, treated 58 chronic wounds of various origins lasting from several months to several years with a balanced solution of salts, amino acids, a high-molecular-weight D-glucose polysaccharide, and ascorbic acid, and reported, in small and medium-sized wounds with ages of 5 to 20 years, a healing time of four to eight weeks.

Larger lesions were managed with autografts.

The paper of Bergman et al. [84], cited by Kaufman and only available as an abstract, did not reveal information on the pH of the wounds successfully treated with honey.

## 8. General Questions

### 8.1. What Is the Optimal pH for Optimized Wound Healing from the Cell Perspective?

According to Kruse et al., keratinocytes were demonstrated to have optimum vitality at a pH of around 7.5 [85], while fibroblasts’ best viability was observed at pH 7.5 to 11.

Sharpe et al. [86] found that the optimal pH for keratinocyte and fibroblast proliferation migration was 8.55, and that for cell proliferation, between 7.2 and 8.3, where the expression of cytokeratin K1, which is significant for differentiated keratinocytes, and K5, significant for basal keratinocytes, was studied. The optimal pH for keratinocyte migration from explants was 8.55, while optimal active proliferation was observed between pH 7.58 and 8.55. There was no difference between fibroblasts and keratinocytes in proliferation. The pH range that was tolerated by keratinocytes was wider than that for fibroblasts. No difference between the cells expressing K1 or K5 could be found. The optimal pH for fibroblast attachment to tissue culture plastic was 8.06, and that for keratinocytes was 8.3, after 24 h.

Lönnqvist et al. describe that at pH-5, there is no, with pH 6, there is moderate, and with pH 7, there is normal re-epithelization in vitro [87].

### 8.2. What Is the Optimal Take-Rate of Skin Grafts with Respect to the pH?

Chai et al. [88] investigated the pH of granulation tissue concerning skin grafts and stated that pH values of 7.2 to 7.5 resulted in the best take rates. Wounds with a pH lower than 6.5 had <10^7^ *Staph. aureus* or *Escherichia coli* CFUs. Wounds with a pH of 8.0 showed *Pseudomonas aeruginosa* at more than 10^8^ CFUs.

Sayegh described the correlation of pH with graft take in 24 ulcers of different origins, with a table describing the take rates according to pH (Sayegh et al., 1988b). Cesny described the maximal skin graft take as occurring at pH 6.8 to 7.4 (Congress report, animal studies).

In 1956, Richard [89] described the take rate for 90 granulating wounds. In 12 cases transplanted at a pH of 6.8 to 7, the average take rate for the skin grafts was 0.1%; in 14 with a pH of 7.0 to 7.2, it was 6%; in 10 with pH 7.2 to 7.4, it was 47%. In 18 with a pH of 7.4, it was 91.4%, and in 36 with a pH above 7.4, it was 99%.

### 8.3. What about the Killing Ability of Leukocytes Dependent on pH?

In 1997, Allen et al. [90] investigated the respiratory burst activity, i.e., oxygen consumption and superoxide production, over ranges of 30 to 300 mm of PO_2_, 0 to 40 mmol/L of glucose, and pH 6 to 8. The oxygen consumption and production and oxidant generation correlated with the oxygen tension of the incubation fluid. They stated that the leukocyte killing capacity was substantially impaired at low oxygen concentrations, including at lower pH although to a lesser extent. Increased oxygen tension at the wound site also augmented the bacterial killing capacity of leukocytes in the wound. The reason for this is that peroxide production is dependent on the NADPH-linked oxidase. The oxidant generation was also dependent on pH, temperature, and glucose concentration, but less so.

### 8.4. What Is the Relationship of Matrix Metalloproteinases and pH?

Matrix metalloproteinases are classified according to their function as collagenases, gelatinases, stromelysins, matrilysins, enamelysins, membrane-type MMPs, and others.

Matrix metalloproteinases are pH- and temperature-dependent for their efficacy and stability. MMP1, classified as Collagenase is one of the first acting MMPs in wound repair and has the highest efficacy at a pH between 6 and 7 [91]. Gelatinases have maximal activity at a pH of 8.6 [92] against marine psychrophile *Vibrio* and 7.2 against *Streptococcus faecalis* [93]. Matrilysin (MMP7) has a broad optimum pH range of 5.0–9.0 [94].

## 9. Results

### 9.1. Topical Lactate Application

Lactate was applied in different forms, as a polyglactin [38], as a cream applied topically [69], as a Matrigel delivering lactate [35,70], as an implant in lactide polymers [95], and by the external application of polylactide membranes [74,96,97].

The implantation of polyglactin resulted in a statistically significant increase in VEGF and a doubling of TGF-β levels; IL-1β was significantly elevated on Day 7, but on the other days, it showed no significant difference from controls. The topical application of creams showed effects in the range of 1.5 to 3% but not at 5%, at which apoptosis and cell death occurred. Delivery by Matrigel demonstrated that different lactate–glycolide preparations could be used for controlled lactate release. The context of lactate-dependent angiogenesis exhibited a direct relationship between VEGF and lactate in a non-toxic range, and oxamate could antagonize lactate’s effect. Implants of lactate polymers resulted in an increase in angiogenesis and functional vessel density. Delivery using polylactic membranes resulted in an increase in telomerase activity and the absolute skin cell count, a reduction in systemic oxidative stress, an early decrease in IL-6 and TNF-α, and a time-limited increase in TGF-β. The topical application of lactate resulted in an increase in VEGF and angiogenesis in a dose-dependent manner within a non-toxic range, a reduced systemic inflammatory response, and reduced oxidative stress, with positive effects on wound healing.

### 9.2. Topical Acidification

Wounds have a lower oxygen saturation owing to the damage to vessels. When exposed to air, they lose CO_2_ and become alkalotic. Bacterial contamination may increase ammonia production. “Acidification” was tested regarding the ammonium-binding capacity of acidic applications and their effects on wound healing under different pH conditions by Leveen et al. [75] with Carbopol, Aserbine, acetic acid, and different concentrations of polyacrylic solutions. All the applications showed an initial drop in pH for a limited time. The release of the acids seemed to be the influencing component, which was the shortest in acetic acid and Aserbine, while the polymeric acids seemed to exhibit concentration-dependent effectivity. Wound healing failure was attributed to an alkaline pH in the wound.

By the continuous irrigation of partial-thickness burns with buffered solutions at different pH levels using irrigation disks over a week, they found that the acidic- and normal-pH-treated wounds healed faster, and after three weeks, they exhibited a higher healing rate than the controls, which still showed incomplete healing. The scar thickness was increased in the alkalinity group [77]. The addition of amino acids and ascorbic acid to balanced solutions was described as successful [83].

Topical acidification showed positive healing effects, which could be mainly attributed to the reduction of the strongly alkaline pH to a more physiological one, avoiding levels that are cytotoxic to keratinocytes and fibroblasts and inhibiting the activity of MMPs.

### 9.3. The Optimum pH for Wound Healing

Table 1 shows the optimum pH for cells to enable wound healing results from in vitro testing. The values seem to be essential for spontaneous wound healing without grafting.

Fibroblasts showed optimal vitality, proliferation, and attachment at pH 7.5 to 8.55, remaining vital up to a pH of 11, while keratinocytes showed the best results for vitality, proliferation, migration, and epithelialization at a pH of 7 to 8.55. Thus, the optimal pH for wound healing seems to be in the range of 7.5 to 8.5.

### 9.4. The Optimum Wound pH for Grafting

The effect of pH on grafted wounds was determined by registering the take rates for grafts at different pH values. In addition, the kind and severity of the bacterial contamination of wounds might influence the pH, as described by Chai, who found a pH lower than 6.7 in patients with high counts of *Staph. albus* or *Escherichia coli* and pH > 8 in patients with *Pseudomonas aeruginosa*. Table 2 shows the results of the optimum pH for wound grafting. 

### 9.5. What Is the Optimum pH for the Bacterial Killing Capacity of Leukocytes?

When measuring the peroxide production of neutrophils, a lower oxygen concentration and a lower pH reduced the superoxide production in leukocytes. As Allen demonstrated in his Figure 6, the peroxide production at a pH of 7 was about five times higher than that at pH 6. The optimal killing capacity was demonstrated to occur between pH 7 and 7.5 [90].

## 10. Discussion

### 10.1. MMPs and Biofilms

The literature on different approaches to improving wound healing was reviewed with an emphasis on the pH of the wound, oxygen saturation improvement, and improvement of the metabolic situation. However, the authors are aware that, recently, components influencing wound healing such as MMPs and biofilms have increasingly been topics of research. The growing knowledge has brought about a better understanding of the immensely complex network of MMPs and TIMPs. Unfortunately, initial studies on the binding of the Zn^++^ necessary for enzyme action did not result in therapeutic applications [98]. Nevertheless, this and other approaches are expected to be evaluated in the future [99], although most research is devoted to cancer.

Interestingly and independently from MMPs and biofilms, papers from earlier years described success in treating chronic wounds. For example, clinical applications of acidic dressings [77,78,79,83] and the application of topical lactate [35,38,69,70], as well as measures to increase the local oxygen saturation, such as occlusive dressings or HBO treatment [1], resulted in shorter healing times. Fortunately, most of these methods remain feasible but became lost from the therapeutic toolbox. In this paper, several approaches were discussed.

### 10.2. Correcting the pH:

When evaluating the effect of acidic dressings, the main mechanism might be the conversion of a highly alkalotic pH in chronic wounds to a less alkalotic one, possibly reducing MMP activity [100], making the oxygen delivery to the wound higher than that in a strongly alkalotic one. The higher oxygen delivery and lactate result in increased vasculogenesis and enhance the bacterial killing capacity of leukocytes in a neutral environment. Chronic wounds are marked by excessive activity of MMPs breaking down the extracellular matrix. According to Trengove et al. and Schultz et al., protease activity decreases during healing. Therefore, decreasing the pH might curb protease hyperactivity and contribute to healing. Adjusting the pH from 8 to 4 was associated with an 80% reduction in protease activity [101,102]. According to their table, when reducing the pH from 8 to 7.5, the reduction in protease activity might be about 25%. It was shown that the application of nanocrystalline silver could reduce MMP-9 levels to a greater extent than silver nitrate [103].

pH levels in chronic wounds are dependent on the stage of the ulcers, as demonstrated by Dissemond et al. Venous ulcers of stage one showed an average of pH 5.7, stage two 6.9, and stage three 7.6. A correlation to the ulcer type could not be established [16]. pH in feet ulcers was inconstant, but was used to diagnose infections [17].

Vu et al. [1] described a device to predict wound healing based on pH measurements.

Nevertheless, it was shown that applying acidifying dressings can correct or at least reduce an alkalotic pH. However, the short duration of the pH adjustment, owing to the short acidity-neutralizing time, may limit the effects of dressings. However, even short-term pH drops may be effective in restoring the capability of fibroblasts and keratinocytes to proliferate and migrate and improve wound healing. Polymeric dressings with controlled degradation to active monomers had the most extended effects on pH [75]. The speed of degradation to the active form determines the concentration of the active component and the duration reservoir of the bioactive polymer. In addition, the composition of the polymers influences the kind of acidic anions released. The application of physiological NaHCO_3_ can correct acidosis. The effect can be monitored easily with a pH probe or litmus paper.

Acidifying can be done by wet dressings soaked with acidic components like lactic acetic acid, citric acid, or others in a not cytotoxic solution [104,105] or polymeric membranes from the lactic acid [106], which has the advantage of a controlled release and antioxidant activity, where it is hypothesized that it might contribute to a reduced grafting rate in partial-thickness burns [73,106]. Controlling the pH before grafting might be highly relevant in healing per the second intention with delayed grafting and dermal templates used in a two-step procedure.

### 10.3. Improving Hypoxia and Lactate Accumulation

Local hypoxia is the consequence of a disturbed vascular network and reduced supply, the increased oxygen demand by the production of reactive oxygen species, and a high metabolic demand for tissue neogenesis as by increased cell division and increased extracellular matrix production [18]. Depending on the depth of a wound, the intradermal capillary plexus is damaged along with papillary arterioles and venules or the intradermal plexus [107]. Hypoxia is the most extensive in the center of the wound and shows a gradient from the margin to the center [29]. Hypoxia can be reduced by external oxygen or by increasing neovascularization, which can be used therapeutically. In addition, the body itself accumulates lactate, indicating increased metabolic demand and action in the inflammatory repair process, fueling the cells necessary to initiate revascularization [81].

Hypoxia increases lactate production and accumulation [19,20]. This lactate accumulation in and around cells lowers the pH, working against the alkalinity derived from the reduced O_2_ partial pressure and CO_2_ loss. Accumulation of lactate triggers the neovascularization [38,42,108]. Hypoxia by itself is not sufficient.

In addition, lactate levels from 5 to 10 mM induce a hypoxia-like response in fibroblasts and other cells dependent on and independent of HIF [109]. With the local vascular network disturbed and the local oxygen tension reduced but still not zero, VEGF increases and initiates angiogenesis and cell growth, and vascular ingrowth. The low oxygen tension stimulates collagen synthesis through TGF-β1 and procollagen [110] and supports the healing process.

With a better vascularization of the wound, the oxygen tension rises, the CO_2_ loss is reduced, and healing is accelerated, by providing oxygen for the other metabolic needs as increased cell division, stabilizing of collagen, and extracellular matrix generation. Capillary sprouting takes place from Day 3 to Day 10 after injury [111]. It is initiated by growth factors binding to their receptors on endothelial cells and dissolving the basal lamina. Activated endothelia are enabled to proliferate and form sprouts. Integrins, as superficial adhesion molecules, help to organize the endothelial cells, which produce matrix metalloproteinases that lyse the surrounding tissue, facilitating endothelial progress. Hyperbaric oxygen is effective in this state in hypoxic wounds [5], possibly by increasing stem cells proliferation, which was found in polylactides as well [20,21]. The combination of both therapeutic approaches might be useful, but had not been described until now.

This process can be supported by topical lactate application.

However, as demonstrated by Trabold, additional topical and implanted lactate significantly increased VEGF and TGF-β levels. Rendl demonstrated the effectiveness of creams containing lactate with VEGF at 1.5 and 3% concentrations, while the effect was diminished at higher concentrations, indicating toxicity, with higher apoptosis and cell death. The composition of the lactate–glycolide application as a powder with external and intraperitoneal application can control the release of the lactate [70]. Lactate from Matrigel induced angiogenesis in a dose-dependent manner. Lactate subcutaneously implanted as polymers induced an angiogenic response with increased vessel density; topically applied lactate as a lactide polymer improved telomerase activity and skin quality in human partial-thickness burn wounds. Lactate reduced systemic oxidative stress parameters and resulted in a shorter healing time under reduced oxidative stress. It also decreased IL-6 and TNF-α early and increased TGF-β for a limited period.

Thus, internal and external lactate can support wound healing by supplying energy to cells in fresh and chronic wounds [112] and act as an antioxidant, reducing oxidative stress, which increases MMP1 production in the presence of LPS and H_2_O_2_ [113]_._ Thus, we can learn from all these studies that topical lactate application improves wound healing and reduces oxidative stress systemically. However, the lactate effect must be stopped in time, to avoid stiffness and contracted scars being produced by myofibroblasts [114].

When there is granulation tissue, the wound can either heal spontaneously or need to be grafted. In spontaneous wound healing, the pH must be between 7.5 and 8.5 to enable cell proliferation and differentiation, and the closure of the wounds. An alkalotic pH can be improved by applying lactate using the most feasible method as a membrane.

If the wound must be grafted, the optimum take rate occurs between pH 6.8 and 7.2 or at pH ≥ 7.4.

The optimum pH for enabling bacterial killing by leukocytes is between 7 and 7.5. Therefore, treatment aims to create a pH that supports skin and fibrous cell proliferation and healing.

## 11. Conclusions

There are simple components in wound treatment that determine its success. First, the metabolic situation for undisturbed wound healing must be established. Lactate fuels cells by imitating hypoxia under normoxic conditions and encourages metabolic processes such as vasculogenesis and angiogenesis, improving the vascularization and local oxygen tension. Lactate is the key to normalizing the oxygen tension, the acceleration of wound healing, and initiating metabolic processes in fresh wounds and “dead” ulcers, as demonstrated in chronic wounds by Nischwitz et al. [115]. A normally vascularized wound after lactate treatment is less prone to infections, which has been demonstrated for pigskin.

The control and regulation of the wound pH with adequate dressings or topical measures can improve wound healing, prevent the chronification of wounds of all etiologies, and restart the healing process.

## 12. Additional Information on Request

Registered products releasing lactate from membranes available on the market for the use in skin and wounds are Suprathel^®^ and Supra SDRM^®^ from Polymedics Innovations GmbH, Denkendorf, Germany. Other resorbable, lactate releasing products are available for Orthopedic Surgery.

## Figures and Tables

**Table 1:** Optimal pH for cells.

Literature (First Author)	Fibroblasts	Keratinocytes	Function Tested	Kind of Testing
Kruse	7.5–11	7.5	Vitality	In vitro
Sharpe		8.55	Migration	Explants in tissue cultures
Sharpe	7.58–8.55	7.58–8.55	Proliferation	
	8.5	8.3	Attachment	
Lönnqvist		5	No epithelialization	In vitro
		6	Moderate epithelialization	
		7	Normal epithelialization	

**Table 2:** Optimum wound pH for grafting from literature.

Study (First Author)	Optimal Take Rate at pH	Method	Remarks
Chai	7.2–7.5	Human	
Sayegh	7.2 (*n* = 2)	Animal study, *n* = 15	
	7.2 (Take rate 90%), *n* = 2	Human, *n* = 25 deep second or 3rd degree burns	
	>7.4 (*n* = 18)	Chronic ulcers *n* = 24	Total loss in pH < 7.4
Cesny	6.8 to 7.4		
Richard	>7.4 (Take rate: 99%)		No takes between 6.8 to 7

## Data Availability

No new data were created or analyzed in this study. Data sharing is not applicable to this article.
